# First Assessment of Risk Factors for *Giardia* spp. Infection in Hospitalized Patients from Romania

**DOI:** 10.3390/microorganisms14010062

**Published:** 2025-12-26

**Authors:** Rodica Georgiana Dărăbuş, Marius Stelian Ilie, Diana Maria Dărăbuş, Gheorghe Dărăbuş, Tudor Rareş Olariu

**Affiliations:** 1 Discipline of Parasitology, Department of Infectious Diseases, “Victor Babes” University of Medicine and Pharmacy, 300041 Timisoara, Romania; georgiana.darabus@umft.ro (R.G.D.); rolariu@umft.ro (T.R.O.); 2 Center for Diagnosis and Study of Parasitic Diseases, Department of Infectious Disease, “Victor Babes” University of Medicine and Pharmacy, 300041 Timisoara, Romania; 3 Discipline of Parasitology, Faculty of Veterinary Medicine, University of Life Sciences “King Mihai I” from Timisoara, 300645 Timisoara, Romania; gheorghe.darabus@fmvt.ro; 4 Oftalmo Sensory-Tumor Research Center—ORL (EYE-ENT), Ophtalmology Department, “Victor Babes” University of Medicine and Pharmacy, 300041 Timisoara, Romania; 5 Patogen Preventia, 300124 Timisoara, Romania

**Keywords:** *Giardia* spp., Romania, human prevalence, risk factors

## Abstract

This study aimed to identify and evaluate the risk factors associated with *Giardia* spp. infection among generally hospitalized patients with various conditions specific to infectious diseases in Western Romania. A total of 312 patients, ranging in age from 2 months to 90 years and originating from both urban and rural settings, were included in the analysis. Fecal specimens were collected and analyzed using the Rapid-VIDITEST Crypto Giardia antigen test, a qualitative chromatographic assay for the detection of *Giardia* spp. Infection-related risk factors were assessed through a standardized questionnaire completed by adult patients or by the guardians of pediatric patients. The overall prevalence of *Giardia* spp. infection was 4.8%. Of the risk factors analyzed, only age demonstrated a statistically significant association with infection status (*p* < 0.05). Notably, the highest prevalence (12.5%) was recorded in the <1-year age group (2–11 months), with a marked decrease in prevalence observed among patients aged ≥60 years. Other evaluated risk factors—including area of residence, gender, contact with animals, pet ownership, hand hygiene after animal contact, type of housing (house or apartment), fruit washing practices, use of potable water, use of public transportation, international travel, and visits to playgrounds or swimming pools—did not show a statistically significant association with *Giardia* spp. infection among the study participants. The lack of statistical significance for several expected factors may be related to limited statistical power resulting from the low number of positive cases. These findings contribute to the current understanding of *Giardia* spp. transmission dynamics and may inform future research efforts aimed at elucidating relevant risk factors. Furthermore, the results may support the development of targeted public health interventions (focusing on infants and their caregivers) and prevention strategies.

## 1. Introduction

*Giardia duodenalis* (syn. *Giardia lamblia* and *Giardia intestinalis*) is a leading cause of parasitic diarrheal disease worldwide. It represents a major public health concern, imposing significant economic burdens and contributing to global morbidity and mortality. According to the World Health Organization (WHO) [1,2], approximately one-third of the global population is infected with intestinal parasitic diseases (IPDs), with most cases occurring in children. Transmission primarily occurs via the fecal–oral route, with contaminated water or food, as well as direct contact with infected individuals or animals, serving as a common source of infection.

*G. duodenalis* is classified into eight genetic assemblages (A–H) based on host specificity and molecular variation. Assemblages A and B are known to infect humans, and certain sub-genotypes within these groups exhibit zoonotic potential. Within the genus *Giardia*, six species have been identified: *Giardia duodenalis* (also known as *lamblia* and *intestinalis*), *G. agilis*, *G. muris*, *G. microti*, *G. ardeae*, and *G. psittaci*. Among these, *G. duodenalis* infects a wide range of mammalian hosts and is the etiological agent of giardiasis—one of the most prevalent parasitic enteric diseases [3,4,5].

Several factors contribute to the widespread prevalence of *G. duodenalis*, including its low infectious dose, rapid spread following excretion, and the high environmental resistance of its cysts—particularly in contaminated water and food sources [6]. Companion animals, such as cats and dogs, may also act as zoonotic reservoirs [7].

Globally, over 280 million people are estimated to suffer annually from giardiasis-associated diarrhea, with children in unsanitary environments being particularly vulnerable [3,8]. Clinical manifestations range from profuse, fatty diarrhea and abdominal cramps to nausea and fatigue. However, infections may also be asymptomatic. Chronic giardiasis can disrupt gut homeostasis, leading to changes in the microbiota, villous atrophy, increased intestinal permeability, nutrient malabsorption, and growth retardation. Post-infectious complications may include food allergies, irritable bowel syndrome, and chronic fatigue syndrome. Although *Giardia* infection elicits robust innate and adaptive immune responses, it is often associated with a low-grade inflammatory profile, distinguishing it from many other intestinal pathogens [9,10].

The prevalence of *G. duodenalis* varies across countries due to differences in risk factors. In developing nations, these include limited education [11], inadequate access to treated drinking water [12,13], unsafe water storage [14,15], poor hand hygiene [14,16,17], lack of proper sanitation [18], rural residency [19], poverty, household crowding, and substandard food hygiene [18,19,20,21]. In contrast, in developed countries, institutionalization, homosexuality, international travel, and immigration have been identified as key risk factors [22,23]. Additionally, independent variables such age [24,25] and seasonal variation [14] may also influence infection rates.

There is little information regarding giardiasis in the human population from Romania. The few available data are based only on prevalence surveys conducted among institutionalized children or their medical staff [26,27]. However, no studies concerning the risk factors for human giardiasis were previously performed. Therefore, in this study, we decided to assess the prevalence and risk factors for *Giardia* infection among hospitalized patients from Western Romania.

## 2. Materials and Methods

### 2.1. Samples and Data Collection

In 2024, a total of 312 fresh fecal samples were collected using sterile stool containers and promptly transported to the laboratory for analysis. These samples were obtained from patients admitted to the Victor Babeș Infectious Diseases Hospital in Timișoara, located in Western Romania. The study population comprised all generally hospitalized patients, with various conditions specific to infectious diseases, between February and April 2024. The only exclusion criterion was refusal to participate; however, no patients declined involvement. Consequently, all eligible individuals during the study period were interviewed and examined. Each participant received medical treatment for the primary condition that necessitated their hospitalization.

### 2.2. Study Population

The study population comprised both male and female patients, with ages ranging from 2 months to 90 years. Participants were stratified into seven age groups: <1 year, 1–5 years, 6–10 years, 11–17 years, 18–39 years, 40–59 years, and ≥60 years. All hospitalized patients resided in either urban or rural areas of Western Romania. At the time of fecal sample collection, some participants exhibited digestive symptoms (diarrhea), while others were asymptomatic. In our study, symptomatic versus asymptomatic was defined only in relation to gastrointestinal signs, specifically the presence or absence of diarrhea at the time of fecal sample collection. Was included this variable in the analysis and compared the prevalence of *Giardia* between participants with diarrhea and those without diarrhea as part of the risk-factor assessment. This comparison is clinically relevant because *Giardia* infection can be associated with diarrhea but may also occur without overt gastrointestinal signs, and restricting attention to symptomatic individuals could underestimate circulation and transmission.

Animal exposure (household pet ownership/contact—yes/no and farm animal contact—yes/no) varied among individuals, ranging from direct ownership of animals to incidental contact with various animal species not owned by the participants.

### 2.3. Laboratory Tests

The diagnosis of human giardiasis was performed using rapid tests. The Rapid-VIDITEST Crypto Giardia is a qualitative chromatographic immunoassay test for the simultaneous detection *Cryptosporidium* and *Giardia* antigen in human feces specimens. This test has a sensitivity of 95.6% (95% CI, 84.9–99.5%) and a specificity of 97.5% for *Giardia* (95% CI, 91.4–99.7%) (Rapid-VIDITEST, VIDIA spol.s r.o., Nad Safinou II 365, 252 50 Vestec, Czech Republic). The procedure was performed according to the manufacturer’s instructions.

### 2.4. Risk Factors

To assess potential risk factors associated with infection, all study participants—or the parents or legal guardians in the case of pediatric patients—were asked to complete a detailed, structured questionnaire. This instrument was designed to gather epidemiologically relevant information across several domains. Specifically, the questionnaire included items related to:

- Demographic characteristics, including area of residence (urban or rural) and gender;
- Clinical information, such as the presence or absence of digestive symptoms;
- Animal exposure, including ownership of domestic pets, direct or indirect contact with animals;
- Hygiene practices, particularly handwashing behavior following contact with animals;
- Water and food safety, including the source and treatment of drinking water, and habits related to the washing of fruits prior to consumption;
- Environmental and recreational exposure, including visits to swimming pools and children’s playgrounds;
- Public transportation usage;
- International travel history, including the occurrence of travel outside the country.

The aim of the questionnaire was to comprehensively document potential environmental, behavioral, and demographic risk factors that could be associated with infection dynamics in the study population.

The questionnaire was not previously validated in a comparable sample, and was an ad hoc tool developed by a multidisciplinary team comprising epidemiologists, physicians, veterinarians, and infectious disease specialists. Its design was informed by established guidelines and protocols from the European Centre for Disease Prevention and Control (ECDC) [28], particularly those pertaining to the surveillance and risk assessment of giardiasis. This methodological approach ensured that the questionnaire was both scientifically robust and aligned with international standards for evaluating risk factors associated with *Giardia* infection. The inclusion of expertise from diverse disciplines further enhanced the questionnaire’s validity and comprehensiveness in capturing relevant epidemiological and environmental data.

### 2.5. Statistical Analyses

Statistical analyses were conducted using Epi Info™ software, version 7.2.6 (Centers for Disease Control and Prevention, Atlanta, GA, USA, 2023). The Fisher Exact and Mantel–Haenszel tests (two-tailed) was applied to evaluate the statistical significance of associations between potential risk factors and *Giardia* infection. For the initial univariate associations we applied Fisher’s exact test (two-tailed) for 2 × 2 contingency tables. Additionally, the Mantel–Haenszel stratified procedure was also used in the univariate screening, but the results were not statistically significant, so the tables show the results of the first test. A *p*-value ≤ 0.05 was considered indicative of statistical significance.

In addition, multivariate logistic regression analyses were performed to control for confounding variables and assess the strength of associations. These analyses were carried out using the Stats Models library in Python version 3.9 and IBM SPSS Statistics, version 20.0 (IBM Corp., Armonk, NY, USA). All statistical procedures were conducted using validated and widely accepted methods, and the results, including their interpretation, were thoroughly reviewed and confirmed by the authors to ensure accuracy and reliability.

### 2.6. Ethical Approval

The study was conducted in accordance with the principles outlined in the Declaration of Helsinki and was approved by the Ethics Committee of the Victor Babeș University of Medicine and Pharmacy, Timișoara, Romania (Approval No. 12/17 March 2023). Written informed consent was obtained from all participants prior to their inclusion in the study. For participants under the age of 18, consent was obtained from a parent or legal guardian.

## 3. Results

### 3.1. Age and Gender

*Giardia* spp. was detected in all age groups, except for those aged 6–10 years and 11–17 years (Table 1).

The highest prevalence of *Giardia* spp. was observed in the <1-year age group (12.5%). A statistically significant difference in prevalence was found among individuals aged ≥60 years (*p* < 0.05). The youngest positive case was a 4-month-old female infant from an urban area who lived in a house and presented with diarrhea at the time of sample collection. The oldest infected individual was a 76-year-old woman from a rural area, also living in a house, who exhibited febrile symptoms. No significant difference in *Giardia* spp. prevalence was observed between males and females.

### 3.2. Environmental and Lifestyle Risk Factors

The prevalence of infection was not significantly different between participants living in urban areas (5.0%) and those in rural areas (4.7%) (Table 2).

Although the prevalence of *Giardia* spp. was higher among individuals living in houses (6.2%) compared to those living in apartments (2.0%), the difference was not statistically significant. None of the three institutionalized individuals included in the study tested positive for *Giardia* spp. Among the 312 patients, 168 reported experiencing diarrhea within the past six months, and Giardia infection was detected in 11 of these individuals (11.5%). However, some *Giardia*-positive patients did not report any episodes of diarrhea.

Most patients reported consuming potable water; however, *Giardia* spp. was detected in 12 of them (6.1%). Some patients—both infected and non-infected—were unsure about the potability of their water supply or whether it had been tested.

When asked about fruit-washing practices prior to consumption, most patients (224; 96.3%) reported doing so regularly. *Giardia* spp. was detected in both individuals who consistently washed fruits and those who did so only occasionally or not at all. Most patients (79.5%) reported infrequent use of swimming pools; however, *Giardia* spp. was still detected within this group. A lower prevalence of infection was observed among patients who reported visiting children’s playgrounds. While only a small proportion of participants used public transportation (13.8%), *Giardia* spp. infection was more frequently detected among this subgroup. Few participants had traveled abroad, yet *Giardia* spp. infection was identified in both travelers and non-travelers.

## 4. Discussion

Limited information regarding human giardiasis in Romania is currently available to the international medical community. This study represents the first report assessing risk factors for *Giardia* spp. infection in Western Romania. The survey evaluated multiple potential risk factors among hospitalized patients admitted to two departments of the Infectious Diseases Hospital in Timișoara. The overall prevalence of *Giardia* spp. infection was 4.8%. This prevalence may be influenced by the presence of various underlying medical conditions among the study population, which could act as potential risk factors by increasing individual susceptibility to *Giardia* spp. infection.

A similar study conducted in northwestern Romania reported a *Giardia* spp. prevalence of 2.82% among hospitalized patients [29]. Higher prevalence rates have been recorded in low-income countries, including Iran (10.6%) [30], Pakistan (17%) [16], Nepal (18.9%) [31], Ecuador (12.6%) [32], Brazil (17.3%) [33], Algeria (14.6%) [34], and Mozambique (44%) [35].

Regarding differences among age groups, taking the <1-year category as the reference group—which exhibited the highest prevalence (12.5%)—a statistically significant difference (*p* < 0.05) was observed when compared to the ≥60-year age group, which showed a prevalence of 2.7%. However, in two age groups (6–10 years and 11–17 years), *Giardia* spp. infection was not detected in any of the patients, making it impossible to calculate the odds ratio for these categories.

The high prevalence observed among infants under one year of age may be attributed to primary infections that contribute to reduced immune resistance. This is supported by findings from a previous study indicating that children are 17.9 times more susceptible to *Giardia* infection compared to adults and that the risk of coinfection is higher among immunocompromised individuals [25]. However, our results contradict those of Fakhri et al. (2021) [24], who reported that age does not play a significant role as a risk factor for *Giardia* infection. Nonetheless, other authors, such as Krumrie et al. (2022) [36], have identified outbreaks associated with diaper-wearing children attending outpatient settings, which is consistent with the four young children included in our study. A recent study conducted among children in Nicaragua reported a higher incidence of *Giardia* infection in the 12–24-month age group [37]. These differences may reflect the influence of distinct risk factors, as well as variations in study methodologies. The findings of the present study are consistent with data reported by the World Health Organization, which estimates that approximately 450 million children and infants are infected with *Giardia* and similar intestinal parasites worldwide [1,2].

The prevalence of *Giardia* spp. infection appeared to be higher among male patients compared to females; however, the difference was not statistically significant, suggesting that gender cannot be considered a risk factor in this context. Similarly, Fakhri et al. (2021) [24] concluded that neither age nor gender showed a significant association with *Giardia* infection.

The prevalence of *Giardia* spp. infection observed in this study showed no statistically significant differences between patients from urban and rural areas. In contrast, a study conducted in Argentina reported a higher prevalence of *Giardia* infection in peri-urban settings [38]. Another study from Ethiopia, conducted among elementary school students aged 6–21 years, reported a *Giardia* prevalence of 13.2%, with rural residence identified as a significant risk factor. This association was linked to poor hygiene practices, such as lack of handwashing before meals, inadequate nail hygiene, improper use of latrines, and limited family education [39]. Moreover, increased prevalence in rural areas has also been attributed to the potential for zoonotic transmission [40]. In contrast to these findings, our study did not identify any residence-related differences in *Giardia* spp. infection prevalence.

From an epidemiological perspective, both in rural and urban areas of developing countries, sociocultural factors—such as socioeconomic and educational levels, as well as hygiene practices—are recognized as key determinants of parasitic infections in general, and *Giardia* infection in particular [41].

Although the prevalence of *Giardia* spp. infection was three times higher among patients living in houses compared to those residing in apartments, the difference was not statistically significant. It is possible that living conditions, particularly more frequent contact with the outdoor environment, may have contributed to the increased risk of infection. Conversely, other researchers [42] have reported higher prevalence and intensity of endoparasite infections—including *Giardia*—in areas dominated by apartment buildings. Their explanation suggests that dogs living in houses are typically confined to private yards with limited exposure to public spaces, whereas dogs in apartments are usually walked at least once daily by their owners. In addition, higher human and pet population densities in apartment-dense neighborhoods may contribute to increased environmental contamination, particularly in green areas surrounding these buildings.

Some studies have demonstrated a higher prevalence of subclinical *Giardia* infection (i.e., without overt symptoms) among infected individuals [43]. However, Alemu et al. (2023) [44] reported that children without current diarrhea but with a history of diarrhea within the past month had higher odds of testing positive for *Giardia* compared to children without such a history. In our study, the difference in *Giardia* spp. prevalence between patients with and without diarrhea was not statistically significant. Nevertheless, the prevalence among patients with diarrhea (6.5%) was more than double that of patients without diarrhea (2.8%). This finding should be interpreted in the context of the study population, the majority of whom were undergoing antibiotic therapy at the time of sample collection.

Based on a systematic review and meta-analysis by Tawana et al. (2023) [45], which included studies conducted in Algeria, India, Libya, and Madagascar, the prevalence of *Giardia* spp. was found to be 12.3% among individuals with diarrhea and 9.7% among those without diarrhea. In contrast, the same review reported higher prevalence rates among asymptomatic individuals in studies from Thailand, Bangladesh, Mozambique, and Tanzania. These differences have been attributed to variations in individual immune responses to *Giardia* infection.

Several studies in the scientific literature have indicated that water can serve as a source of *Giardia* infection [46,47,48]. In the present study, *Giardia* spp. infection was identified among patients who reported consuming potable water or who were unsure about the potability of their drinking water, but not among the smaller group of patients who reported consuming non-potable water. However, the differences in infection prevalence among these three categories were not statistically significant.

Parasitic infections represent a significant public health concern, as they can lead to complications such as behavioral disturbances, growth retardation, weight loss, anemia, and other health issues. A systematic review and meta-analysis by Fakhri et al. (2021) [24] concluded that the prevalence of giardiasis is influenced by multiple risk factors, including lack of access to safe drinking water, inadequate hand hygiene, sex, age, limited access to education, and absence of proper sanitation facilities.

It is well established that fruits and vegetables provide essential nutrients and minerals important for human health; however, they can also serve as vectors for pathogenic organisms. The contamination of raw fruits and vegetables with human pathogenic parasites, including *Giardia*, poses a global public health threat despite the recognized health benefits of these foods [49]. In the present study, *Giardia* spp. infection was identified only among patients who reported washing their fruits regularly or occasionally. Notably, none of the 18 patients who reported never washing fruits tested positive for *Giardia* infection. According to some authors, such as Zeynudin et al. (2024) [50], fruits and vegetables from peri-urban areas appear to be more heavily contaminated and may represent a significant source of infection in humans.

Although several authors [18,20,21] have identified multiple factors contributing to the increased prevalence of giardiasis, in our study, some of these potential risk factors—such as fruit washing, swimming pool visits, use of public transportation, and visits to children’s playgrounds—did not show statistically significant associations with *Giardia* infection.

International travel was not found to have a statistically significant impact on the prevalence of *Giardia* spp. infection in our study. However, a higher proportion of positive cases was observed among patients who reported occasional international travel. In this context, several authors have acknowledged the increased susceptibility to giardiasis among international travelers [22,23].

While the prevalence of giardiasis was slightly higher among patients who had contact with animals, the difference was not statistically significant. Similarly, pet owners exhibited a higher prevalence of *Giardia* infection, though this difference also lacked statistical significance. When different animal groups were analyzed separately in relation to *Giardia* spp. infection among their owners, no statistically significant associations were observed. However, higher infection rates were noted among patients who owned cats and dogs.

These findings suggest that certain animal species may represent potential risk factors for *Giardia* spp. infection. In fact, two groups of patients who owned birds, and birds in combination with sheep, showed even higher infection prevalence than those who owned dogs and cats. The presence of *Giardia* spp. in galliform birds and sheep has been confirmed in previous studies [51,52]; however, less is known about their zoonotic potential and the role they may play in human transmission.

Handwashing, as a fundamental hygiene practice, is recognized as an important preventive measure against *Giardia* infection [24,53]. However, some patients in this study reported washing their hands only occasionally or not at all after contact with animals. This may partially explain the higher infection prevalence (7.8%) observed among those who practiced hand hygiene inconsistently. Nevertheless, the differences in *Giardia* spp. prevalence between individuals who adhered to proper hand hygiene and those who did not were statistically significant.

Overall, this epidemiological evaluation presents certain limitations, as it was conducted on patients from a single medical institution, with a relatively small sample size in some age groups and relied on a single diagnostic approach. The lack of significance of several anticipated risk factors (diarrhea, rural residence, poor hygiene) is probably due to the low overall prevalence (4.8%) and sample size (*n* = 312), which may be another limitation. Despite these constraints, the findings contribute valuable insights into the epidemiology of diseases and may inform the implementation of effective preventive strategies.

## 5. Conclusions

This study provides the first evaluation of risk factors associated with *Giardia* spp. infection in hospitalized patients in Western Romania. The overall prevalence of *Giardia* infection was 4.8%, with the highest infection rate observed in infants under one year of age. Age was the only statistically significant risk factor identified, highlighting the particular vulnerability of this group, possibly due to immature immune responses and increased exposure to primary infections.

Although higher prevalence rates were observed in males, rural residents, pet owners, and patients with diarrhea, the differences were not statistically significant. Similarly, potential risk factors such as type of housing, use of potable water, international travel, handwashing habits, and contact with animals did not show significant associations with infection status. Interestingly, *Giardia* was more frequently detected in patients who reported irregular fruit washing, occasional handwashing after animal contact, and inconsistent hygiene practices, although these trends were not statistically significant either.

The findings align with global studies indicating a wide range of contributing factors to giardiasis prevalence, including water and food safety, hygiene behavior, and animal contact [18,20,21,50]. While previous studies have emphasized the potential zoonotic role of companion animals, our data suggest a possible association (a numerical, non-significant association) between *Giardia* infection and ownership of birds and sheep, although further research is needed to assess the zoonotic potential of these species.

Overall, these results underscore the complexity of *Giardia* transmission dynamics and suggest that no single factor fully accounts for the observed prevalence. The study highlights the need for broader, multicenter investigations with larger sample sizes, along with molecular studies, to better understand the epidemiology and zoonotic potential of *Giardia* spp. in Romania and similar settings. Enhanced public health education on hygiene and food safety may also help reduce the burden of this infection.

## Figures and Tables

**Table 1:** Distribution by age and gender of positive cases with *Giardia* spp.

Variable		Number of Tested Patients *n* = 312	Number of Patients with Positive Test Results *n* = 15 (%)	OR 95%CI	*p*-Value
Age group (years)	<1	32	4 (12.5)	Ref.	
	1–5	64	2 (3.1)	4.428 (0.765–25.614)	0.093
	6–10	18	0	Undefined	0.282
	11–17	4	0	Undefined	1.000
	18–39	38	4 (10.5)	1.214 (0.278–5.299)	1.000
	40–59	43	2 (4.7)	2.928 (0.501–17.092)	0.392
	≥60	113	3 (2.7)	5.238 (1.108–24.763)	0.042
Gender	Female	166	7 (4.2)	0.759 (0.268–2.148)	0.609
	Male	146	8 (5.5)		

Legend: OR = Odds ratio; CI = Confidence interval; Ref. = reference.

**Table 2:** Prevalence and associated risk factors of *Giardia* spp. infection in humans from Romania ascertained by questionnaire.

Variables		Number of Tested Patients *n* = 312	Number of Patients with Positive Test Results *n* = 18 (%)	OR 95%CI	*p*-Value
Area of residence	Rural	193	9 (4.7)	0.921 (0.319–2.656)	1.000
	Urban	119	6 (5)		
Housing	House	210	13 (6.2)	Ref.	
	Apartment	99	2 (2)	3.200 (0.708–14.465)	0.156
	Institutionalized	3	0	Undefined	1.000
Digestive symptoms in the last 6 months	Yes	168	11 (6.5)	2.452 (0.763–7.976)	0.183
	No	144	4 (2.8)		
Potable water source	Yes	196	12 (6.1)	Ref.	
	No	26	0	Undefined	0.368
	Don’t know	90	3 (3.3)	1.891 (0.520–6.875)	0.403
Fruits washed before consumption	Yes	224	11 (4.9)	Ref.	
	No	18	0	Undefined	1.000
	Sometimes	70	4 (5.7)	0.852 (0.262–2.765)	0.760
Frequenting swimming pools	Yes	22	1 (4.5)	Ref.	
	No	248	13 (5.2)	0.860 (0.107–6.906)	1.000
	Sometimes	42	1 (2.4)	1952 (0.116–32.798)	1.000
Frequenting children’s playgrounds	Yes	83	1 (1.2)	Ref.	
	No	185	12 (6.5)	0.175 (0.022–1.375)	0.070
	Sometimes	44	2 (4.5)	0.256 (0.022–2.906)	0.275
Using public transport	Yes	43	3 (7)	Ref.	
	No	206	11 (5.3)	1.297 (0.346–4.857)	0.717
	Sometimes	63	1 (1.6)	4.536 (0.456–45.128)	0.303
Frequenting travel abroad	Yes	26	0	Ref.	
	No	270	13 (4.8)	Undefined	0.613
	Sometimes	16	2 (12.5)	Undefined	0.139

Legend: OR = Odds ratio; CI = Confidence interval; Ref. = reference.

## Data Availability

The original contributions presented in this study are included in the article. Further inquiries can be directed to the corresponding authors.
